# Improvement of Mechanical Strength of Tissue Engineering Scaffold Due to the Temperature Control of Polymer Blend Solution

**DOI:** 10.3390/jfb12030047

**Published:** 2021-08-14

**Authors:** Azizah Intan Pangesty, Mitsugu Todo

**Affiliations:** 1 Department of Metallurgical and Material Engineering, Faculty of Engineering, Universitas Indonesia, Depok 16424, Indonesia; 2 Research Institute for Applied Mechanics, Kyushu University, Kasuga, Fukuoka 816-8580, Japan; todo@riam.kyushu-u.ac.jp

**Keywords:** polymer blend, pre-heat treatment, porous scaffold, PCL/PLCL blend, mechanical properties

## Abstract

Polymeric scaffolds made of PCL/PLCL (ratio 1:3, respectively) blends have been developed by using the Thermally Induced Phase Separation (TIPS) process. A new additional technique has been introduced in this study by applying pre-heat treatment to the blend solution before the TIPS process. The main objective of this study is to evaluate the influence of the pre-heat treatment on mechanical properties. The mechanical evaluation showed that the mechanical strength of the scaffolds (including tensile strength, elastic modulus, and strain) improved as the temperature of the polymer blend solution increased. The effects on the microstructure features were also observed, such as increasing strut size and differences in phase separation morphology. Those microstructure changes due to temperature control contributed to the increasing of mechanical strength. The in vitro cell study showed that the PCL/PLCL blend scaffold exhibited better cytocompatibility than the neat PCL scaffold, indicated by a higher proliferation at 4 and 7 days in culture. This study highlighted that the improvement of the mechanical strength of polymer blends scaffolds can be achieved using a very versatile way by controlling the temperature of the polymer blend solution before the TIPS process.

## 1. Introduction

Tissue engineering provides a promising strategy to regenerate or reconstruct damaged soft tissues (e.g., muscles, skin, heart valves, blood vessels, etc.). Natural polymers including collagen, fibrin, and elastin have been widely explored to develop scaffolds for application in soft tissue engineering [1,2,3,4]. Collagen, for example, is a good candidate for skin and blood vessels graft due to excellent biocompatibility, low immunogenicity, and good cell adhesion [5,6,7]. However, those natural polymers such as collagen often degrade rapidly [8,9], which potentially causes the tissue-engineered graft to collapse before the mature tissue is formed. Moreover, the mechanical properties are often much lower than the targeted tissues which limited their application to some extent [10,11,12].

Compared to natural polymers, synthetic degradable polymers provide tailorable mechanical and degradation properties [13]. Due to its rubbery properties [13], Poly ε-caprolactone (PCL) has been extensively used for soft tissue engineering scaffolds, such as vascular [14], urethra [15], and cardiac patch [16]. It degrades around 50% in 4 years [17], resulting in compromisingly non-toxic products in the human body. However, due to its long degradation time, PCL is usually physically blended or copolymerized with other polymers. Physical blending provides a more feasible route to modify the degradation time and the mechanical properties than copolymerization. In a previous study, PCL was blended with poly-(lactide-co-ε-caprolactone) (PLCL) to develop a porous tubular scaffold at optimum ratio 1:3 (respectively) by the TIPS method [18]. Unfortunately, the tensile mechanical strength of the blend scaffold is lower than that of the neat PCL and the neat PLCL scaffold. The tensile mechanical strength of the neat PCL, neat PLCL, and PCL/PLCL (1:3) scaffolds were 171 kPa, 434 kPa, and 147 kPa, respectively. Phase separation in polymer blends can create spherulites or islands morphology in continuous matrix that affects the macroscopic mechanical properties, as reported by [19,20].

A recent study reported that annealing at elevated temperature as a post-treatment process improved crystallinity and tensile strength of the electrospun-poly (glycolide-co-lactide) [21]. However, the pre-heat treatment process on polymer blends solutions has been little studied. In this study, a strategy to improve the mechanical properties of the PCL/PLCL blend scaffold was addressed by applying the pre-heat treatment to the blend solution before the TIPS process. The temperature of the blend solution was varied at 20, 30, 40, 50, and 60 °C. The immiscibility of the polymer blend scaffold was analyzed by using Fourier Transform Infrared Spectroscopy (FTIR) and the thermal property was evaluated by using Differential Scanning Calorimetry (DSC). After the freeze-drying process, the effect of temperature variation on the microstructural and mechanical properties was evaluated by using Field Emission Scanning Electron Microscope (FE SEM) and Universal Testing Machine, respectively. The in vitro cell proliferation test was also performed to examine the cytocompatibility of the polymer blend scaffolds.

## 2. Materials and Methods

### 2.1. Pre-Heat Treatment on the Blend Solutions

PCL (Mw 530,000 g/mol) (CelgreenH7, Daicel Corporation, Osaka, Japan) and PLCL 70:30 (Mw 410,000 g/mol) (Gunze ltd., Kyoto, Japan) pellets at a ratio of 1:3 respectively, were dissolved in 1,4-dioxane (Kishida Chemical, Osaka, Japan) solvent with a final concentration of 6% (*w*/*v*). This specific ratio of blend solution is used based on our previous study that results in the optimum ratio for rebound properties [18]. The blend solutions in glass vials were subsequently heated in the oven to five different temperatures (20, 30, 40, 50, and 60 °C) for 3 h.

### 2.2. Fabrication of PCL/PLCL Blend Scaffold

The solid-liquid phase separation method was adopted to prepare the tubular scaffold, as mentioned in [22]. A cylindrical rod made of polytetrafluoroethylene (PTFE) (Ø 10 mm) which was pre-cooled in −80 °C for 1 h was immediately immersed into the preheated blend solution and pulled out at a constant rate of 100 mm/min. The sample was stored again at −80 °C for another 2 h, then was dried at −50 °C for 24 h using a freeze-drying machine (Tokyo Rikakikai, Tokyo, Japan). Finally, the tubular scaffold was removed by pulling it out from the mold and was then stored in a dehumidifier chamber ready for further use.

### 2.3. Solvent Casting Method

The PCL/PLCL thin film was prepared by the solution casting method. The blend solution is heated to 20 °C. A total of 1 mL of blend solution was poured into a plastic case. After the solvent completely evaporated, polymer film was formed. The surface morphology was then observed by using FE-SEM to characterize the phase separation behavior. The procedure was repeated to produce the PCL/PLCL film from the 50 °C blend solution.

### 2.4. Fourier-Transform Infrared Spectroscopy (FTIR) Analysis

Infrared analysis (FT/IR-4200, JASCO Corporation, Tokyo, Japan) was performed to confirm the chemical structures of the polymer blend scaffold. The sample size of 2 mg was mounted on the silver plate and IR spectra were recorded at an average of 16 scans in wavenumbers ranging from 600 to 4000 cm^−1^.

### 2.5. Differential Scanning Calorimetry (DSC) Analysis

Thermal properties including melting point (*T_m_*) and glass transition temperature (*T_g_*) were measured by DSC (DSC-60, Shimadzu Corporation, Kyoto, Japan). A sample weight of 2.5 mg was mounted into an aluminum sample pan. The sample was heated at a heating rate of 10 °C/min from room temperature to 230 °C. The temperature and DSC curves were recorded.

### 2.6. Microstructure Observation

The microstructure images were observed by using Field Emission-Scanning Electron Microscope (FE-SEM). A sample size of 10 × 10 mm was prepared. The sample was coated with Pt-Pd using an anion sputter coater (Hitachi, Tokyo, Japan). Both the surface and cross-section of the sample were then observed using FE-SEM (S-4100, Hitachi, Tokyo, Japan).

### 2.7. Measurement of Pore Area and Strut Size

The ImageJ software, a public domain java program provided by The National Institute of Health of United State Government, was used to measure the pore area and strut size of the FE-SEM images that were obtained in Section 2.6, as adopted from [23]. Briefly, the image analysis was done by adjusting the threshold thus the boundaries between the pores and struts can be distinguished clearly. The area of each pore was measured by running the particle analysis command embedded in that software. The average and the distribution of pore size were measured based on three image analyses of each sample. The strut size was measured based on the thickness of the strut surrounding each pore.

### 2.8. Mechanical Testing

**Figure 1:**
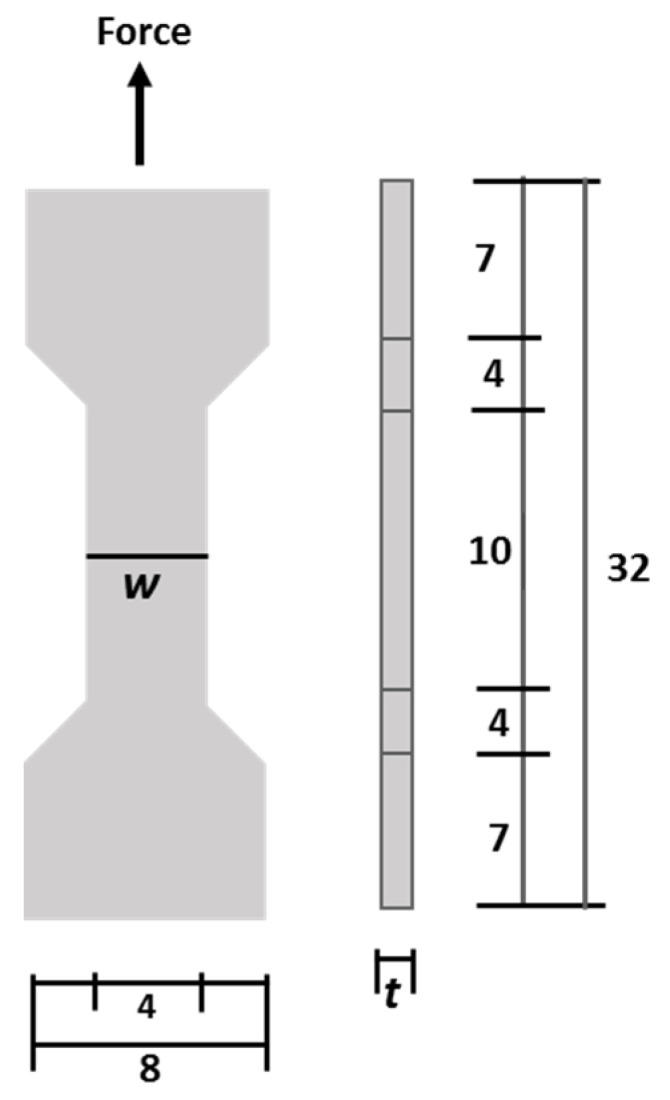
A dog-bone-shaped sample for mechanical testing. All dimensions in mm, *w*: width; *t*: thickness.

Tensile mechanical tests were performed by using a Shimadzu Compact Tabletop Testing Machine (Shimadzu Corporation, Tokyo, Japan)) with a 10 N load cell and a crosshead speed of 1 mm/min. The tubular scaffold was cut into a dog bone shape, as shown in Figure 1. Based on the load-displacement relation that was monitored during the test, the stress (*σ*) and strain (*ε*) were evaluated using the following Formulas (1) and (2), respectively.

(1) $$Stress\;\left(\sigma \right)=\frac{F}{A}=\frac{F}{wxt}$$

(2) $$Strain\;\left(\varepsilon \right)=\frac{\Delta L}{L}$$

where *F* is the force under the tensile test, *A* is the area of the cross-sectional sample which is calculated by width (*w*) × thickness (*t*). *L* is the length of the sample under measurement and Δ*L* is the displacement after loading at each time.

The thickness of the specimen depends on each scaffold produced. The thickness was measured using a digital caliper with an accuracy of ±0.1 mm. The elastic modulus was calculated by identifying the linear region in the resulting stress–strain curve. The tensile strength was calculated based on the ultimate stress before fracture. The strain energy density was calculated based on the area under the ultimate stress using Kaleidagraph (version 4.01, Synergy Software, Reading, PA, USA).

### 2.9. Cell Proliferation Test

Prior to the cell proliferation test, all samples were sterilized by using 70% ethanol and rinsed with Phosphate Buffered Saline (PBS) (Gibco, New York, NY, USA) three times. Human mesenchymal stem cells (hMSCs) (UE6E7TE, Riken Bio Research Center, Kyoto, Japan) were seeded onto a square sample (0.5 × 0.5 cm) with a cell density of 5000 cells/sample and cultured in a cell growth medium containing MEM-α (Wako Chem, Tokyo, Japan), 10% Fetal bovine serum (Corning, Tewksbury, MA, USA), and 1% penicillin-streptomycin (MP Biomedicals LLC., Tokyo, Japan) in an incubator at 37 °C/5% CO_2_. The cell proliferation was evaluated at 4 and 7 days of culture. Briefly, the sample was rinsed using PBS, then immersed in 500 µL of a cell counting kit-8 (CCK-8, Dojindo Laboratories, Kumamoto, Japan) reagent. After the soaked sample was incubated for 1 h at 37 °C, the optical density (O.D.) was measured by using a microplate reader (2040 ARVOTM X2, Perkin Elmer Co., Yokohama, Japan) at 450 nm. The O.D. was converted into the number of viable cells based on a calibration curve.

### 2.10. Statistical Analysis

Statistical analysis was performed using ANOVA with Fisher’s LSD post hoc test (version 4.01, KaleidaGraph, Synergy Software, Reading, PA, USA). Data are represented as mean ± standard deviation (SD) and *p* < 0.05 is considered as statistically significant.

## 3. Results

### 3.1. Pre-Heat Treatment and Fabrication of PCL/PLCL Scaffolds

**Figure 2:**
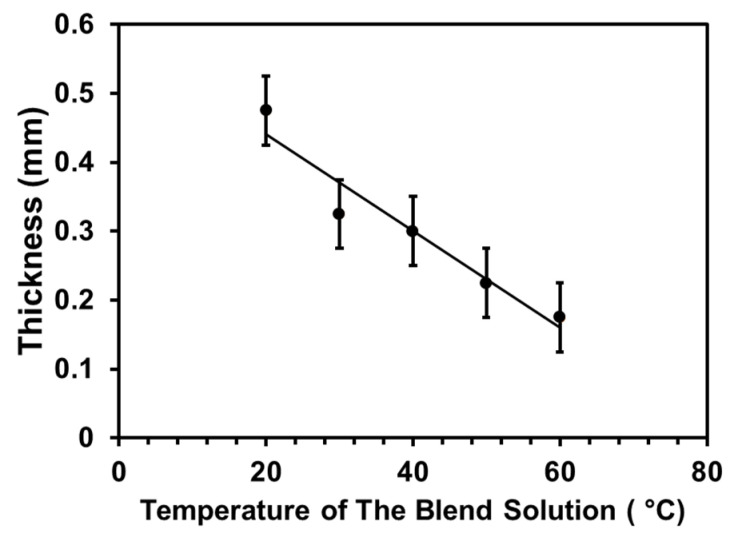
The effect of temperature variation of the blend solution on the thickness of the tubular scaffolds. Data was presented as mean ± SD (*n* = 4).

As mentioned in the fabrication method, the casting into a tubular shape was done by immersing the cylindrical rod vertically into the blend solution. This technique caused the thickness of the tubular scaffold to vary with the tendency of the thickness to increase from top to bottom due to the effect of gravity. The values measured in Figure 2 are the thickness of the middle part of the tubular scaffold.

Temperature variation of the blend solution affected the thickness of the tubular scaffold. As shown in Figure 2, as the temperature increased, the scaffold’s thickness decreased from 0.47 ± 0.05 mm to 0.17 ± 0.05 mm at 20 and 60 °C, respectively. It should be noted that the thickness represented here was measured using a digital caliper of accuracy ±0.1 mm.

### 3.2. FTIR and DSC Analysis

**Figure 3:**
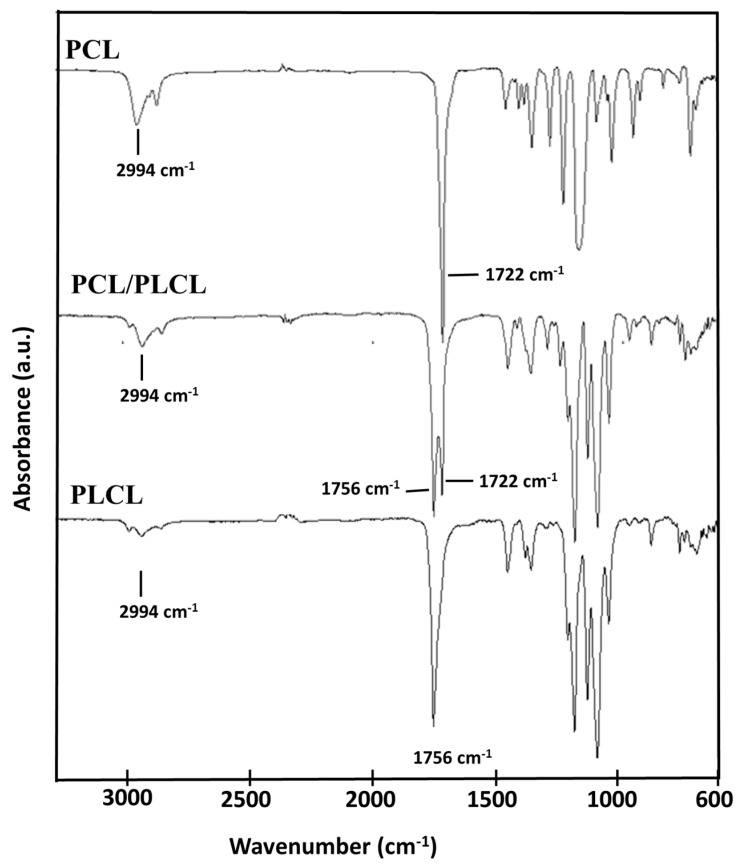
FTIR spectra of PCL, PLCL, and PCL/PLCL scaffold prepared from the 20 °C polymer solution.

In this study, the FTIR and the DSC analyses were performed on the tubular scaffolds prepared from the polymer solutions at 20 °C. The FTIR spectra are shown in Figure 3. Pure PCL has typical peak absorptions at 1722 cm^−1^ corresponding to the carbonyl (C=O) stretching vibration and at 2994 cm^−1^ corresponding to the C–H stretching from the aliphatic chain of Caprolactone (CL). Pure PLCL shows a peak absorption at 1756 cm^−1^ corresponding to carbonyl stretching and a low peak absorption intensity at 2994 cm^−1^, because the composition of CL was less than that of Lactide. The FTIR spectrum of the PCL/PLCL blend shows peak combinations of PCL and PLCL and no new peak is observed. This indicated that both polymers were not covalently linked and only formed physical interactions.

**Figure 4:**
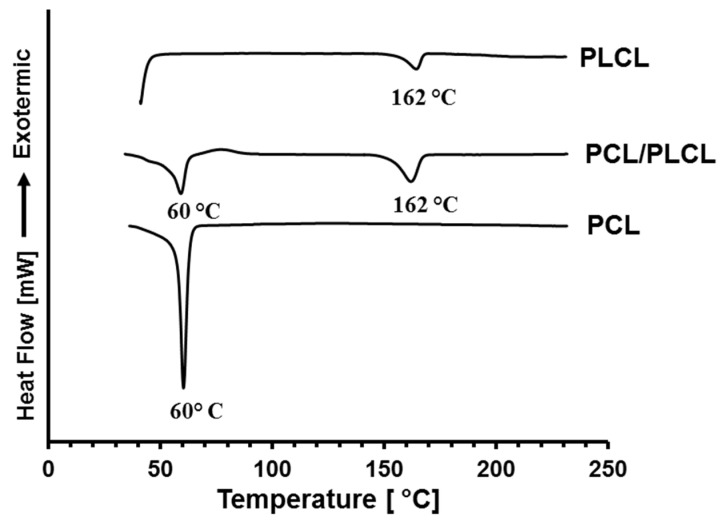
DSC thermograms of PCL, PLCL, and PCL/PLCL blends prepared from the 20 °C polymer solution.

Figure 4 shows the thermal properties of PCL, PLCL, and PCL/PLCL blend scaffolds characterized by using DSC. DSC thermogram of neat PLCL exhibits single endothermic melting points at 162 °C and the melting point of neat PCL is observed at 60 °C. Two endothermic melting points at 60 and 162 °C, are observed in the PCL/PLCL blend thermogram, indicating that the two phases of polymers existed in the blend.

### 3.3. Temperature Variation of the Blend Solutions

#### 3.3.1. Effect on Microstructural Behaviour

**Figure 5:**
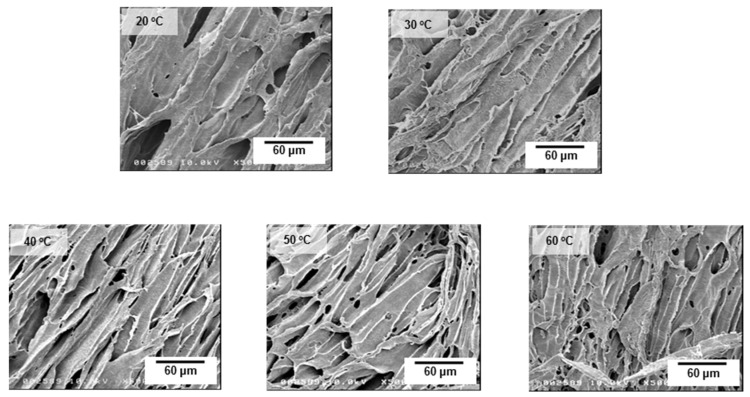
Cross-sectional FE-SEM images of the PCL/PCL scaffold prepared from the various temperatures of the blend solutions.

Figure 5 shows FE-SEM images on the cross-section of the polymer scaffold. All PCL/PLCL scaffolds exhibited laminar pores connected to each other.

**Figure 6:**
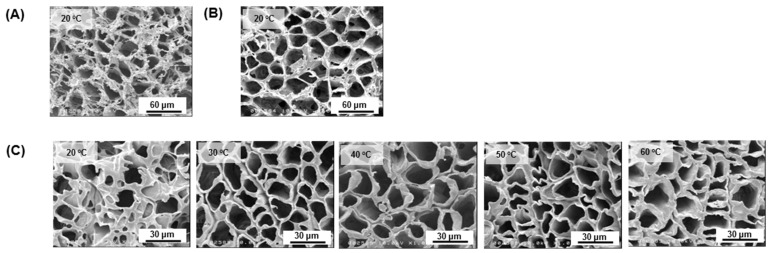
FE-SEM images of the surface morphology of a porous scaffold made of (**A**) neat PCL prepared from pure PCL solution at 20 °C, (**B**) neat PLCL prepared from pure PLCL solution at 20 °C, and (**C**) PCL/PLCL blends as the temperature of the blend solution was increased to 20, 30, 40, 50, and 60 °C.

Figure 6 shows the microstructure behavior of the tubular scaffolds. All of the PCL/PLCL scaffolds obtained from the various temperatures (20, 30, 40, 50, and 60 °C) of the blend solution exhibited porous structures. Neat PCL and neat PLCL scaffolds prepared from the 20 °C polymer solution were used as control groups. It was observed that the PCL scaffold (Figure 6A) showed fibrous struts compared to the PLCL scaffold (Figure 6B). Although the blend scaffolds (Figure 6C) had a composition of 25% of PCL, their strut morphology exhibited non-fibrous structures, similar to that of the neat PLCL scaffold.

**Figure 7:**
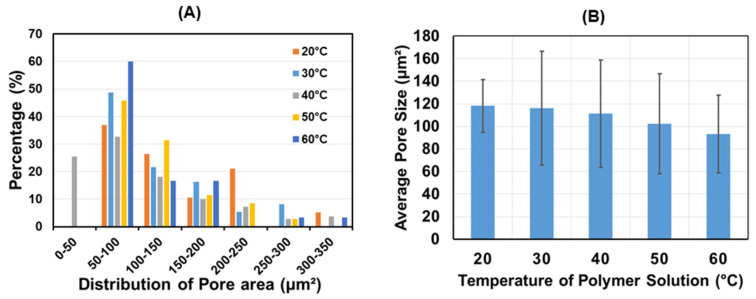
(**A**) Distribution of pore size and (**B**) average of pore size of PCL/PLCL scaffolds prepared at various temperatures of blend solutions (*n* = 35).

Based on FE-SEM images obtained, the pore distribution and the average pore size of the PCL/PLCL scaffolds were calculated, as shown in Figure 7. The pore distribution showed that the pore area of all scaffolds ranged from 50 to 350 μm^2^ with the majority of pore sizes in the range of 50 to 100 μm^2^. The trend of the mean pore size (Figure 7B) decreased with increasing temperature from 118 ± 23 μm^2^ (at 20 °C) to 93 ± 34 μm^2^ (at 60 °C) although no significant difference was observed.

**Figure 8:**
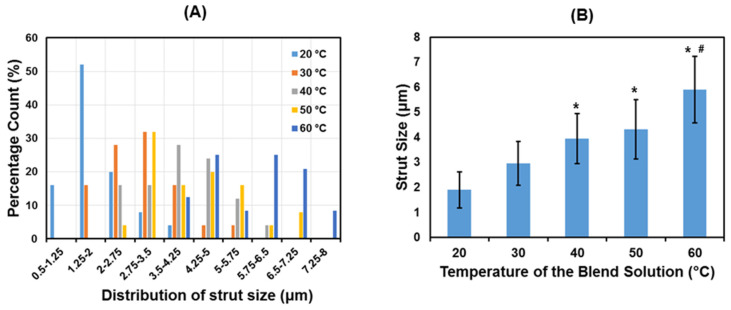
(**A**) Distribution of strut size and (**B**) average strut size of PCL/PLCL scaffolds prepared from various temperatures of blend solutions. *n* = 25; * indicates *p* < 00.5 vs. 20 °C; ^#^ indicates *p* < 00.5 vs. 30 °C.

Figure 8A,B shows the distribution of the strut size and the average strut size of PCL/PLCL scaffolds. The strut distributions (Figure 8A) shift towards a bigger size as the temperature of the blend solution increases. The average pore size (Figure 8B) also tends to increase significantly from 1.89 ± 0.71 to 5.9 ± 1.3 μm.

**Figure 9:**
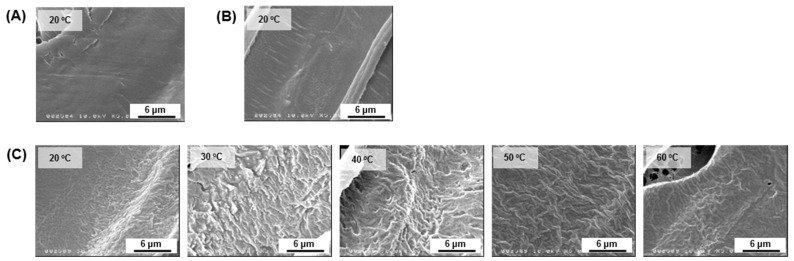
FE-SEM images of the strut surface of a porous scaffold made of (**A**) neat PCL, (**B**) neat PLCL, and (**C**) PCL/PLCL blends with the temperature variations of the blend solution at 20, 30, 40, 50, and 60 °C.

FE-SEM images at higher magnifications showed a smooth surface on the scaffold made of neat PCL (Figure 9A) and neat PLCL (Figure 9B). The surface became rougher on the scaffold blends, however, it tended to be smoother as the temperature of the blend solutions increased, as shown in Figure 9C.

**Figure 10:**
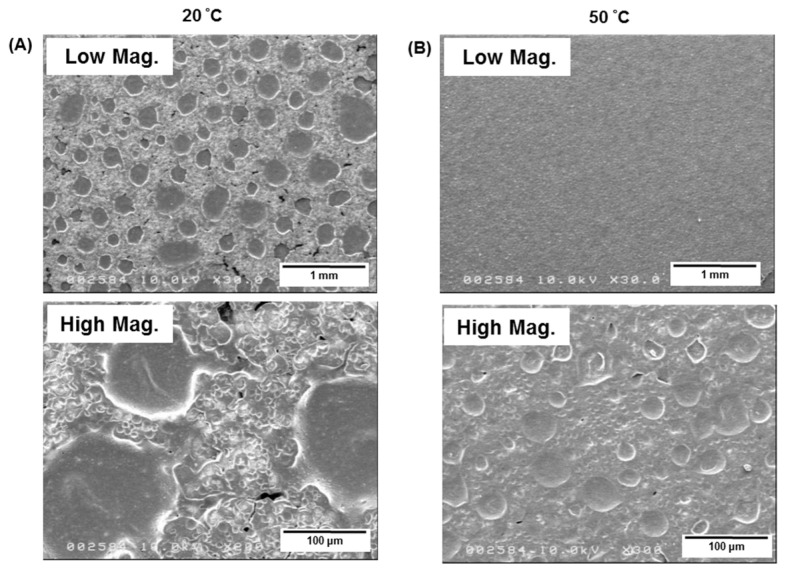
FE-SEM images of PCL/PLCL blend film prepared from the blend solutions at (**A**) 20 °C and (**B**) 50 °C by the solvent casting method.

Phase separation morphology was observed on the PCL/PLCL films prepared by the solvent casting method. As seen in Figure 10, spherical structures are observed in the continuous matrix. Since the blend composition is dominated by PLCL (ratio of PCL/PLCL 1:3), those spherical structures are most likely PCL, and the continuous matrix is PLCL. The average diameter of spherulites prepared at 20 and 50 °C were 213 ± 69 μm and 30.6 ± 6.8 μm, respectively.

#### 3.3.2. Effect on Mechanical Properties

**Figure 11:**
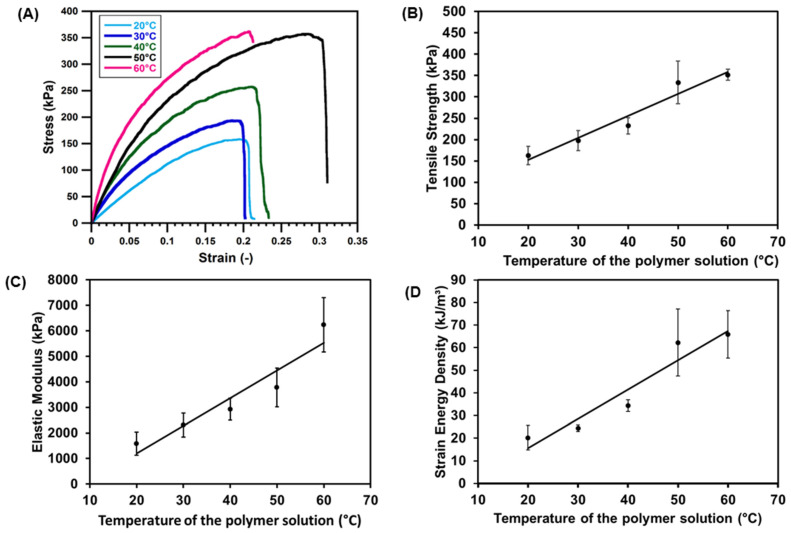
Effects of the temperature variation of the blended solution on the mechanical properties of porous scaffolds. (**A**) Stress–strain curve; (**B**) ultimate tensile strength; (**C**) elastic modulus; (**D**) strain energy density. All data were presented as mean ± SD (*n* = 3).

Mechanical properties of the blend scaffolds with the effect of temperature variation are shown in Figure 11. The stress–strain curve (Figure 11A) indicates a steeper curve as the temperature of the blended solutions increased. The ultimate tensile strength (Figure 11B) improved by more than two-fold higher, from 162 to 417 kPa, when the temperature successively increased from 20 to 60 °C, respectively. Elastic moduli (Figure 11C) significantly improved to 7372 kPa at 60 °C. The increase in elastic moduli means that the material’s stiffness is also increased. The strain energy density (SED) (Figure 11D) also tends to increase with the increased temperature of the blend solutions.

### 3.4. Cell Proliferation

**Figure 12:**
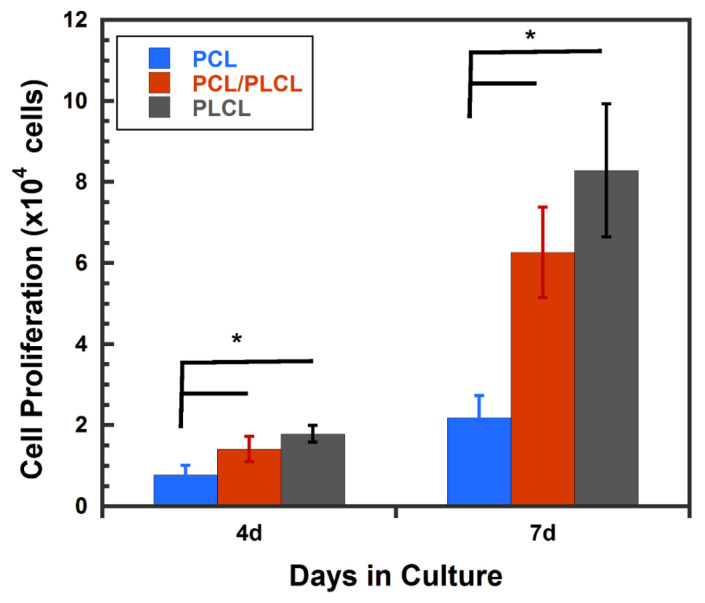
hMSCs proliferation on the porous scaffold made of PCL, PLCL, and PCL/PLCL blends at 4 and 7 days of culture. Data are presented as mean ± SD (*n* = 3), * p < 0.05.

The cytocompatibility test was performed by evaluating the hMSCs proliferation on the PCL, PLCL, and PCL/PLCL scaffolds prepared from 20 °C polymer solution at 4 and 7 days of culture. The hMSCs proliferation on both PLCL and PCL/PLCL scaffolds did not show significant differences during 4 and 7 days in cultures. However, the number of cells on both scaffold types was significantly higher (*p <* 0.05) than that of the PCL scaffold, as shown in Figure 12.

## 4. Discussion

The TIPS technique used to fabricate the tubular scaffold in this study is based on the change in temperature to induce the de-mixing of polymer solution [24]. When a pre-cooled cylindrical rod (−80 °C) was immersed in a polymer solution, it caused the polymer solution to freeze on the surface of the pre-cooled rod. As the result, the rod was coated with the frozen polymer solution. Since the dipping time and polymer concentration were constant, the thickness of the coated polymer was then affected merely by the temperature of the polymer solution. As the temperature of the solution increased, the portion of polymer solidified on the rod was decreased, thus reducing the thickness (Figure 2).

The decrease in scaffold thickness was accompanied by the increase in strut size (Figure 8), which might indicate that density also increased. As a result, the mechanical strengths including elastic moduli, tensile strength, and strain energy density improved as the temperature of the blend solution increased (Figure 11). According to the correlated study, the most effective way to improve the mechanical strength of the porous scaffold was to increase the strut size, as previously reported by [25,26]. The decrease in pore size is also beneficial for strengthening the mechanical properties, as reported by [27]. However, our study showed that the mean pore size (Figure 7) was not significantly decreased with the increase in the blend solution’s temperature. Therefore, the increase in mechanical strength in this study was more influenced by the increase in strut size than the pore size.

The increase in mechanical strength was also associated with changes in the phase separation morphology in PCL/PCL blends. In Figure 9C, rough surfaces are observed at lower temperatures (20, 30, and 40 °C), meanwhile smoother surfaces are found at higher temperatures (50 and 60 °C). The changes in microstructures were also clearly observed in the PCL/PLCL blend film (obtained by solvent casting method) where the PCL created a spherical shape in the continuous structure of PLCL (Figure 10). At 20 °C, the average spherical size was 213 μm. Meanwhile, the mean spherical diameter greatly decreased to 30.6 μm at 50 °C. The decreasing size of spherulites suggested the debonding between PCL spherulites and PLCL matrix was suppressed during tensile fracture, resulting in the increase in the strain energy density as well as the ultimate tensile strength. As reported in [19,20], the decreasing size of spherulites in polymer blends corresponded to the rapid increase in fracture energy.

The cytocompatibility test was performed on the PCL and PCL/PLCL scaffolds prepared from the 20 °C polymer solution. The PCL scaffold had a significantly lower cell proliferation rate than PCL/PLCL scaffolds at 4 and 7 days in culture (Figure 12). This result suggests that PLCL is more favorable for cell growth compared to PCL, although the exact reason for the increased cell growth on PLCL is still unclear. It is known that PCL has a linear alkyl chain that contributes to its hydrophobicity [28], meanwhile PLCL, though it also contains an alkyl chain from the caprolactone backbone, copolymerization with lactide make it less hydrophobic [13]. The difference in surface wettability between PCL and PLCL [29] may contribute to the increase in cell proliferation. However, further study is required to address this finding.

## 5. Conclusions

In the present study, the PCL/PLCL blend scaffolds have been developed and the influence of the temperature control of the blend solution on the mechanical and microstructural behavior was evaluated. It was concluded that:

1. The mechanical strength (including elastic modulus, tensile strength, and strain energy density) increased as the temperature of the blend solution increased;
2. The increase in mechanical strength corresponded to the increase in strut size;
3. The difference in temperature of the blend solution also caused a difference in phase separation morphology. The size of spherulites decreased as the temperature increased;
4. The PCL/PLCL blend scaffold showed more favorable surfaces for cell growth than the neat PCL scaffold.

This study highlighted that the improvement in mechanical strength of polymer blend scaffolds can be achieved using the very versatile method for controlling the temperature of the blend solution before the TIPS process.

## References

[1] Sell S.A., Wolfe P.S., Garg K., McCool J.M., Rodriguez I.A., Bowlin G.L. (2010). The Use of Natural Polymers in Tissue Engineering: A Focus on Electrospun Extracellular Matrix Analogues. Polymers.

[2] Melnick M., Bixler D., Yao L., Swartz D.D., Gugino S.F., Russell J.A., Andreadis S.T. (2005). Fibrin-Based Tissue-Engineered Blood Vessels: Differential Effects of Biomaterial and Culture Parameters on Mechanical Strength and Vascular Reactivity. Tissue Eng..

[3] Swartz D., Russell J.A., Andreadis S.T. (2005). Engineering of fibrin-based functional and implantable small-diameter blood vessels. Am. J. Physiol. Circ. Physiol..

[4] Vázquez J.J., Martínez E.S.M. (2019). Collagen and elastin scaffold by electrospinning for skin tissue engineering applications. J. Mater. Res..

[5] Sundar G., Joseph J., John A., Abraham A. (2021). Natural collagen bioscaffolds for skin tissue engineering strategies in burns: A critical review. Int. J. Polym. Mater..

[6] Wolf K., Alexander S., Schacht V., Coussens L.M., von Andrian U.H., van Rheenen J., Deryugina E., Friedl P. (2009). Collagen-based cell migration models in vitro and in vivo. Semin. Cell Dev. Biol..

[7] Chevallay B., Herbage D. (2000). Collagen-based biomaterials as 3D scaffold for cell cultures: Applications for tissue engineering and gene therapy. Med Biol. Eng. Comput..

[8] Toledano M., Asady S., Toledano-Osorio M., García-Godoy F., Serrera-Figallo M.-A., Benítez-García J.A., Osorio R. (2020). Differential Biodegradation Kinetics of Collagen Membranes for Bone Regeneration. Polymers.

[9] Calciolari E., Ravanetti F., Strange A., Mardas N., Bozec L., Cacchioli A., Kostomitsopoulos N., Donos N. (2018). Degradation pattern of a porcine collagen membrane in an in vivo model of guided bone regeneration. J. Periodontal Res..

[10] Dong C., Lv Y. (2016). Application of Collagen Scaffold in Tissue Engineering: Recent Advances and New Perspectives. Polymers.

[11] Przybysz M., Hejna A., Haponiuk J., Formela K. (2019). Structural and Thermo-Mechanical Properties of Poly(ε-caprolactone) Modified by Various Peroxide Initiators. Polymers.

[12] Al Habis N., El Moumen A., Tarfaoui M., Lafdi K. (2018). Mechanical properties of carbon black/poly (ε-caprolactone)-based tissue scaffolds. Arab. J. Chem..

[13] Kwon I.K., Kidoaki S., Matsuda T. (2005). Electrospun nano- to microfiber fabrics made of biodegradable copolyesters: Structural characteristics, mechanical properties and cell adhesion potential. Biomaterials.

[14] Mo X., Weber H.-J., Ramakrishna S. (2006). PCL-PGLA Composite Tubular Scaffold Preparation and Biocompatibility Investigation. Int. J. Artif. Organs.

[15] Zhang K., Fu Q., Yoo J., Chen X., Chandra P., Mo X., Song L., Atala A., Zhao W. (2017). 3D bioprinting of urethra with PCL/PLCL blend and dual autologous cells in fibrin hydrogel: An in vitro evaluation of biomimetic mechanical property and cell growth environment. Acta Biomater..

[16] Asadpour S., Yeganeh H., Ai J., Kargozar S., Rashtbar M., Seifalian A., Ghanbari H. (2018). Polyurethane-Polycaprolactone Blend Patches: Scaffold Characterization and Cardiomyoblast Adhesion, Proliferation, and Function. ACS Biomater. Sci. Eng..

[17] Ikada Y., Tsuji H. (2000). Biodegradable polyesters for medical and ecological applications, Macromol. Rapid Commun..

[18] Pangesty I.A., Todo M. (2017). Preparation and Characterization of porous tubular scaffold made of PCL/PLCL blends for vascular tissue engineering. J. Mech. Eng..

[19] Park J.-E., Todo M. (2011). Compressive mechanical properties and deformation behavior of porous polymer blends of poly(ε-caprolactone) and poly(l-lactic acid). J. Mater. Sci..

[20] Takayama T., Todo M. (2006). Improvement of impact fracture properties of PLA/PCL polymer blend due to LTI addition. J. Mater. Sci..

[21] Zong X., Ran S., Fang D., Hsiao B.S., Chu B. (2003). Control of structure, morphology and property in electrospun poly(glycolide-co-lactide) non-woven membranes via post-draw treatments. Polymers.

[22] Pangesty A.I., Arahira T., Todo M. (2016). Characterization of Tensile Mechanical Behavior of MSCs/PLCL Hybrid Layered Sheet. J. Funct. Biomater..

[23] (2007). Examples of Image Analysis Using ImageJ, Area. https://imagej.nih.gov/ij/docs/pdfs/examples.pdf.

[24] Conoscenti G., La Carrubba V., Brucato V. (2017). A Versatile Technique to Produce Porous Polymeric Scaffolds: The Thermally Induced Phase Separation (TIPS) Method. Arch. Chem. Res..

[25] Min S.H., Jin H.H., Jun B., Park I.M., Park H.C., Yoon S.Y. (2007). Effect of Reaction Conditions on Pore Configuration and Mechanical Property for Porous Hydroxyapatite Prepared by Polymer Sponge Method. Key Eng. Mater..

[26] Fostad G., Hafell B., Førde A., Dittmann R., Sabetrasekh R., Will J., Ellingsen J., Lyngstadaas S., Haugen H.J. (2009). Loadable TiO_2_ scaffolds—A correlation study between processing parameters, micro CT analysis and mechanical strength. J. Eur. Ceram. Soc..

[27] Zhao H., Li L., Ding S., Liu C., Ai J. (2018). Effect of porous structure and pore size on mechanical strength of 3D-printed comby scaffolds. Mater. Lett..

[28] Singh M., Singh R., Dhami M.K. (2020). Biocompatible Thermoplastics as Implants/Scaffold. Ref. Modul. Mater. Sci. Mater. Eng..

[29] Jeznach O., Kolbuk D., Sajkiewicz P. (2019). Aminolysis of various aliphatic polyesters in a form of nanofibers and films. Polymers.

